# Who made the paintings: Artists or artificial intelligence? The effects of identity on liking and purchase intention

**DOI:** 10.3389/fpsyg.2022.941163

**Published:** 2022-08-05

**Authors:** Li Gu, Yong Li

**Affiliations:** Guangzhou Academy of Fine Arts, Guangzhou, China

**Keywords:** artificial intelligence, painting style, art expertise, framing effect, liking, purchase intention

## Abstract

Investigating how people respond to and view AI-created artworks is becoming increasingly crucial as the technology’s current application spreads due to its affordability and accessibility. This study examined how AI art alters people’s evaluation, purchase intention, and collection intention toward Chinese-style and Western-style paintings, and whether art expertise plays a role. Study 1 recruited participants without professional art experience (non-experts) and found that those who made the paintings would not change their liking rating, purchase intention, and collection intention. In addition, they showed ingroup preference, favoring Chinese-style relative to Western-style paintings, in line with previous evidence on cultural preference in empirical aesthetics. Study 2 further investigated the modulation effect of art expertise. Art experts evaluated less favorably (less liking, lower purchase, and collection intentions) AI-generated paintings relative to artist-made paintings, while non-experts showed no preference. There was also an interaction effect between the author and the art expertise and interaction between the painting style and the art expertise. Collectively, the findings in this study showed that who made the art matters for experts and that the painting style affects aesthetic evaluation and ultimate reception of it. These results would also provide implications for AI-art practitioners.

*The development of full AI (artificial intelligence) could spell the end of the human race.*

*— Stephen Hawking*

## Introduction

Artificial intelligence (AI) is impacting humankind in various aspects. In recent years, scientists have been dedicated to generating creative products such as poetry, stories, jokes, music, paintings, and so on. For instance, taking advantage of Generative Adversarial Networks (GAN), Elgammal et al. (2017) built a new system to generate art by learning about styles and deviating from style norms. Astonishingly, human subjects could not distinguish paintings generated by this system from paintings made by contemporary artists (Elgammal et al., 2017). Although the art-generating agent is mature enough to deceive our eyes (for a review, see Cetinic and She, 2021), a more thought-provoking question is whether it could capture our minds.

Many discussions have been held on the value of artworks created by AI (Ploin et al., 2022). Previous studies have focused on comparing AI-created and artist-made artworks such as paintings (Chamberlain et al., 2018; Hong and Curran, 2019; Gangadharbatla, 2021), performing arts (Darda and Cross, 2022b), and music (Moffat and Kelly, 2006). Researchers are interested in the following three important issues: whether observers could distinguish art generated by AI from those made by humans; whether a bias against AI-created artworks exists; and whether art experience plays a role. First, concerning the ability of observers to discern between computer and man-made art, most prior studies showed that observers could not differentiate between computer-generated and man-made art (Chamberlain et al., 2018; Gangadharbatla, 2021; Darda and Cross, 2022b), while Moffat and Kelly (2006) found that participants could differentiate musical pieces composed by a computer from those composed by humans. Second, a bias against AI-generated artworks has been proven in previous studies. For instance, both implicit and explicit biases against computer-generated paintings were found in Chamberlain et al. (2018), that is, participants perceived paintings categorized as computer-generated by them had lower aesthetic value, irrespective of whether they rated or categorized the paintings first. Third, prior research on art expertise and aesthetics has shown that art experts and non-experts appreciate art differently (Hekkert and Van Wieringen, 1996; Leder et al., 2012; Bimler et al., 2019). Researchers demonstrated that art experts gave higher ratings to artworks (Leder et al., 2012) and showed a much higher level of comprehension than beginners (Leder et al., 2004; Mullennix and Robinet, 2018). A few studies have explored the role of expertise in modulating the bias against AI-generated artworks (Moffat and Kelly, 2006; Chamberlain et al., 2018; Darda and Cross, 2022b). Moffat and Kelly (2006) showed that musicians had a heightened bias against computer-generated musical pieces than non-musicians, whereas Chamberlain et al. (2018) found no modulation effect of art education.

Another line of research in empirical aesthetics, including behavioral studies (Belke et al., 2010; Hawley-Dolan and Winner, 2011; Mastandrea and Umiltà, 2016; Mastandrea and Crano, 2019) and neuroimaging studies (Kirk et al., 2009; Silveira et al., 2015), investigated framing effects by exploring how labels and titles influence aesthetic processing and evaluations. For instance, Mastandrea and Crano (2019) demonstrated that artworks said to be created by famous artists were appreciated more than the same artworks attributed to non-famous artists, being judged more interesting and beautiful. Silveira et al. (2015) investigated whether a socially defined context would set a mental frame that modulates the neurocognitive processing of artworks. Participants were presented with identical abstract paintings from the Museum of Modern Art (MoMA) in New York that were labeled as being either from the MoMA or from an adult education center. Higher neural activation was found when they were evaluating artworks from the MoMA than the education center. Kirk et al. (2009) labeled images as either originating from an art gallery or generated by a computer program (Photoshop) and presented images to participants. They found that participants’ aesthetic ratings were significantly higher for stimuli viewed in the “art gallery” than in “computer program” contexts. Overall, these findings indicate that mental frames play a role in aesthetic evaluations.

In addition, while much research has focused on participants’ perceptions of and biases toward AI-generated artworks, several bodies of research explored the ingroup bias in aesthetic evaluations (for a review, see Che et al., 2018). Prior behavioral and neurological evidence consistently indicated cultural preference (ingroup bias) in aesthetic evaluations (Bao et al., 2016; Yang et al., 2019), that is, people showed a tendency to like artworks originating from one’s own culture more than another culture. Individuals may feel a sense of cultural identity and belongingness when looking at artworks from their own culture and therefore gave higher aesthetic ratings compared to those from another culture (Bao et al., 2016). People showed ingroup bias in evaluating artwork, especially when they lack art-related expertise and experience (Mastandrea et al., 2021), which could be accounted for by the uncertainty-identity theory (Hogg, 2007, 2015). The uncertainty-identity theory is an extension of social identity theory that proposes uncertainty reduction as a major driving force behind group and intergroup actions and social identity processes (Hogg, 2007). According to this theory, people try to lessen their feelings of uncertainty about and connection to themselves through group identification, which would promote ingroup bias in behavior and attitudes.

### The present research

In 2018, a painting called Portrait of Edmond Belamy, created by AI, rocked the art world, selling for $432,500 at Christie’s. Art, as an investment, is embedded with financial attributes. It is essential to understand people’s evaluations and ultimate reception of it. Thus, indicators of paintings’ value, such as purchase intention and collection intention, are worth noting, besides the aesthetic rating. For instance, Gangadharbatla (2021) measured purchase intention as well as the evaluation of artworks. Thus, the current studies measured participants’ liking ratings, purchase intentions, and collection intentions.

People’s ingroup bias in the context of AI-generated artworks and the modulation of art expertise warrants greater understanding. We conducted two studies to explore these questions in this study. The aim of study 1 was to explore the influence of the author (AI and human artists) and the style (Western and Chinese) of paintings in the aesthetic evaluations of Chinese participants without art-related experience or expertise. In line with previous research on the bias against artworks created by machine/AI (e.g., Chamberlain et al., 2018) and the framing effect, we expected a bias against AI-generated paintings irrespective of whether they were of Western or Chinese style. Moreover, based on findings in Mastandrea et al. (2021) and uncertainty-identity theory, we predict that people might be uncertain about the AI-generated context and may resort to cultural identity as an art appreciation heuristic, therefore showing a higher preference for Chinese-style than Western-style AI-generated paintings. Together, our first hypothesis (H1) includes (H1a) Chinese participants showed an overall bias against AI-generated paintings irrespective of whether they were Western or Chinese style; (H1b) Chinese participants favored Chinese-style paintings more than Western-style paintings; and (H1c) participants showed a greater ingroup bias in the context of AI-generated paintings.

Previous evidence suggests that people who are interested in art concur in their aesthetic judgments irrespective of their cultural backgrounds (Child, 1965; Iwao and Child, 1966; Iwao et al., 1969). Moreover, previous research showed an ingroup bias for dance, but not for paintings, and also the modulation role of art expertise (Darda and Cross, 2022a). The aim of study 2 was to explore the influence of the author (AI and human artists) and the style (Western and Chinese) of paintings in aesthetic evaluations and whether it would be modulated by art expertise. Our second hypothesis (H2) extends H1 to incorporate the modulation effect of art expertise, and a three-way interaction would be tested. We first focused on the difference between human-artist and AI-created art for experts only (H2a), then the preference of non-experts toward different styles of paintings (H2b), and the difference between experts and non-experts in evaluating AI-generated paintings (H2c). Together, H2 includes (H2a) art experts showed a greater bias against AI-generated paintings irrespective of whether the painting was in Western or Chinese style; (H2b) non-experts favored Chinese-style paintings more than Western-style paintings irrespective of whether the painting was AI-generated or artist-made; and (H2c) art experts evaluated AI-generated paintings lower than non-experts.

## Study 1

Study 1 explored whether the author of paintings (AI and human artists) and art style (Western and Chinese) influence individuals’ perceptions of paintings.

### Materials and methods

#### Design and participants

Study 1 employed a two-factor mixed-subject design, with the author of paintings (AI art and human artists) as the between-subject factor and the art style (Western and Chinese) as the within-subject factor. Study data were collected from wenjuanxing^1^ in China. As a professional survey company that provides online questionnaires and data collection services, Wenjuanxing has 2.6 million registered members on the platform. All participants were assured that the survey was completely anonymous and confidential, and they were informed that there were no right or wrong answers. A total of 106 participants were recruited online, and they all completed the study *via* the Wenjuanxing platform. The online study took approximately 10 min to complete. Participants first completed an online consent form and a question about their background in art. If the participant responded yes to the question “Have you ever received art-related training or worked in art-related areas?” the questionnaire would skip to the end. All participants reported no professional art-related experience in study 1. The average age of participants was 42.35 years (*SD* = 7.41; range 21–50 years), and 39 were identified as men and 67 as women. Most held 4-year college degrees or higher (59.4% had 4-year college degrees, 9.4% had master’s degrees, and 3.8% had doctoral degrees).

#### Stimuli

The stimuli consisted of 12 high-quality digital paintings (6 were Western style and 6 were Chinese style), including landscape pictures, portraits, and abstract drawings. Following previous research (Mastandrea et al., 2021), the proportions and brightness of the stimuli were in accord with the original format of each painting. The painting sizes and resolution in the display were between 18 and 54 cm in height and between 14 and 24 cm in width, with 72 dpi. All paintings were of similar dimensions, except for one Western-style landscape picture. Half of these paintings were randomly selected and presented to participants. All paintings were made by human artists who were acknowledged in the painting area but were not well-known to the popular. A pilot (*N* = 20) was conducted to exclude the confounding effect that these paintings might be recognized especially by art experts. Both non-experts (*N* = 7) and art experts (*N* = 13; majoring in design and art education) reported that they could not recognize the paintings. This study manipulated the author of paintings (AI art and human artists) by describing the paintings based on the participant’s assigned condition before evaluation. In the AI art condition, the participants read a description of the technology used in art and were told that the paintings were generated by “AlphaART” based on learning original paintings. In the artist-made condition, participants were told that the paintings were done by famous artists. Participants were randomly assigned to one of two conditions, with 53 participants in the AI art condition and 53 participants in the human artist condition.

#### Measures

##### Liking

Following Reymond et al. (2020), we measured participants’ liking of a painting with a rating slider displayed below the image, offering the possibility to rate the paintings from 0 to 100 (0 = “not at all,” 100 = “very much”).

##### The willingness to buy and the willingness to collect

This study measured the willingness to buy a scale (I want to buy this painting; The likelihood of my purchasing this painting is high; The probability that I would buy this painting is high; α = 0.96) using a three-item scale adopted from Dodds et al. (1991), and the purchase intention was calculated by averaging scores on these three items. Furthermore, the willingness to collect (I want to collect this painting; I think this painting is worth collecting; α = 0.94) was measured using a two-item scale, and the collection intention was calculated by averaging scores on these two items. For all items, agreement with the statements was assessed on a Likert-type scale, ranging from 1 = totally disagree to 7 = totally agree.

#### Data analysis

Greenhouse-Geisser corrections were applied to repeated measures analysis of variance (ANOVA) analyses. Partial eta-squared (η*_*p*_*^2^) was used as a measure of effect size, with values of 0.01, 0.06, and 0.14 indicating small, medium, and large effects, respectively (Cohen, 2013). Effect sizes were reported using Cohen’s *d*_*z*_ for within-subject comparisons (Lakens, 2013). All *t*-tests were two-tailed. ANOVAs, simple tests, and *t*-tests were performed using IBM SPSS Statistics 22.0 (SPSS Inc., Chicago, IL, United States).

### Results

#### Liking of paintings

**FIGURE 1:**
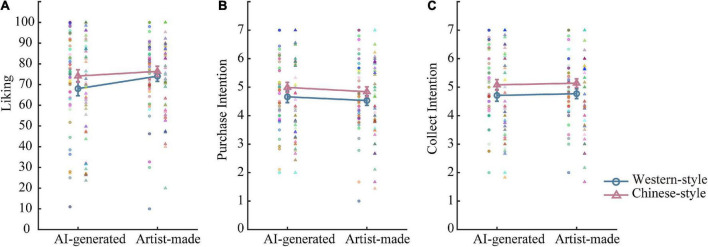
Mean values for the four conditions (author: AI vs. human artists; style: Western vs. Chinese style) in study 1. Participants gave higher ratings for Chinese-style paintings **(A)** and showed higher purchase intention **(B)** and collection intention **(C)** for Chinese-style paintings. Error bars stand for ± S.E.M.

To investigate the effect of the author and style on participants’ liking of paintings, we ran a 2 (author: AI vs. human artists) × 2 (Style: Western vs. Chinese style) ANOVA (refer to Figure 1), with the former as a between-subject factor and the latter as a within-subject factor. The analysis revealed a significant main effect of the painting style [*F*(1, 104) = 9.47, *p* = 0.003, η*_*p*_*^2^ = 0.08], and the main effect of the author and their interaction effect was non-significant. Although the interaction effect was not significant, we conducted *post-hoc* tests (paired-t tests) to verify H1c. Results indicate that the liking of AI-generated Chinese paintings was greater than the liking of AI-generated Western paintings, *t*(52) = 3.45, *p* = 0.001, while no significant difference was found between Chinese and Western paintings made by artists, *t*(52) = 1.11, *p* = 0.272 (refer to Table 1).

**TABLE 1:** Mean values of liking ratings, purchase intention, and collection intention toward paintings in different conditions.

Author	Western-style (M ± SD)	Chinese-style (M ± SD)	Difference (M ± SD)	*t*-value	*P*-value	Cohen’ d_z_
**Liking rating**						
AI-generated	68.03 ± 24.69	74.13 ± 21.69	6.10 ± 12.86	3.45	0.001**	0.47
Artist-made	74.03 ± 18.26	76.35 ± 18.10	2.32 ± 15.22	1.11	0.272	0.15
**Purchase intention**						
AI-generated	4.66 ± 1.69	4.99 ± 1.69	0.33 ± 0.68	3.55	0.001**	0.49
Artist-made	4.53 ± 1.24	4.84 ± 1.26	0.31 ± 1.06	2.1	0.041*	0.29
**Collection intention**						
AI-generated	4.71 ± 1.69	5.08 ± 1.69	0.37 ± 0.69	3.93	<0.001***	0.54
Artist-made	4.77 ± 1.26	5.14 ± 1.10	0.36 ± 1.10	2.42	0.019*	0.33

Post-hoc comparisons between Western-style and Chinese-style paintings are also presented. *Indicates a significance level of p < 0.05. **Indicate a significance level of p < 0.01. ***Indicate a significance level of p < 0.001.

#### Purchase intention and collection intention of paintings

For purchase intention and collection intention, 2 (author: AI vs. human artists) × 2 (style: Western vs. Chinese style) ANOVA tests indicated consistent results. The analysis revealed a significant painting style effect [purchase intention: *F*(1, 104) = 13.54, *p* < 0.001, η*_*p*_*^2^ = 0.12; collection intention: *F*(1, 104) = 17.14, *p* < 0.001, η*_*p*_*^2^ = 0.14], and the main effect of author and interaction effect were non-significant. Paired-t test (refer to Table 1) further indicated that the purchase and collection intention of AI-generated Chinese painting was greater than AI-generated Western painting [purchase intention: *t*(52) = 3.55, *p* = 0.001; collection intention: *t*(52) = 3.93, *p* < 0.001]. Moreover, the purchase and collection intention of artist-made Chinese painting was greater than artist-made Western painting [purchase intention: *t*(52) = 2.10, *p* = 0.041; collection intention: *t*(52) = 2.42, *p* = 0.019].

Results in study 1 showed that the main effect of the author under hypothesis H1a was not significant, suggesting that there was no bias against AI-generated paintings. In addition, the main effect of the painting style was significant, supporting H1b. Chinese participants favored Chinese-style paintings more than Western-style paintings. Although the interaction of the author and the style was not significant, we conducted a *post-hoc* analysis to verify the proposed H1c. Evidence suggests that participants preferred AI-generated Chinese-style paintings to AI-generated Western-style paintings. Specifically, they showed more purchase and collection intentions toward Chinese-style than Western-style paintings, no matter whether the paintings were AI-generated or artist-made. For the liking rating, participants gave a higher rating for AI-generated Chinese-style than Western-style paintings, while no significant preference for artist-made paintings was found.

## Study 2

In Study 2, we further explored the effect of art expertise on painting liking, purchase intention, and collection intention. We recruited participants with art experience from the Guangzhou Academy of Fine Arts (students and teachers majoring in design or art education), which is the only higher art institution in southern China approved by the Ministry of Education. Participants without art experience (non-experts) were recruited from Jinan University in the same city (students and teachers majoring in management). Participants first completed an online consent form and a question about their background in art. If the participant responded yes to the question “Have you ever received art-related training or worked in art-related areas?” they were labeled as art experts, otherwise labeled as non-experts. Participants completed the study online *via* the Wenjuanxing platform, and it took approximately 10 min to complete.

### Materials and methods

#### Design and participants

Study 2 employed a three-factor mixed-subject design, with the art expertise (experts and non-experts) and the author of paintings (AI art and human artists) as the between-subject factors and the art style (Western style and Chinese style) as the within-subject factor. A total of 301 participants were recruited, and 2 participants failed to complete it. Thus, 299 participants were included in the final analysis. The average age of participants was 28.20 years (*SD* = 10.19; range 18–50), and 134 were identified as men and 165 as women. Participants consisted of 143 experts (mean age: 27.34, *SD* = 10.73) and 156 non-experts (mean age: 28.99, *SD* = 9.64), and there was no difference between the two groups in age or education (age: paired *t*-test, *p* = 0.162; education level: Mann-Whitney *U* test, *p* = 0.112). Stimuli, procedure, and measures adopted in study 2 were the same as that in study 1. The reliability of the willingness to buy a scale and the willingness to collect were both over 0.90.

### Results

#### Liking of paintings

For the liking of paintings, we ran a 2 (art expertise: experts vs. non-experts) × 2 (author: AI vs. human artists) × 2 (style: Western vs. Chinese style) ANOVA, with the former two as between-subject factors and the latter as a within-subject factor. The analysis revealed a significant main effect of the author effect [*F*(1, 295) = 8.09, *p* = 0.005, η*_*p*_*^2^ = 0.03], a significant interaction effect of the author and the art expertise [*F*(1, 295) = 3.90, *p* = 0.049, η*_*p*_*^2^ = 0.01], and a significant interaction effect of the style and the art expertise [*F*(1, 295) = 10.42, *p* = 0.001, η*_*p*_*^2^ = 0.03]. Neither the main effect of the style, the main effect of the art expertise, nor the interaction effect of the style and the author, the interaction effect of the three factors were significant (*Fs* < 3.59, *ps* > 0.05).

**FIGURE 2:**
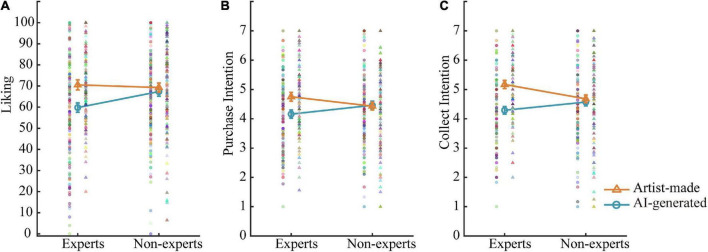
Author (AI and artist) by art expertise (experts and non-experts) interaction. Consistent results were found for liking rating **(A)**, purchase intention **(B)**, and collection intention **(C)**. Error bars stand for ± S.E.M.

The results of the author and the art expertise interaction are presented in Figure 2A, and mean values are listed in Table 2 (left panel). Simple effect analysis further showed that experts showed more liking toward artist-made paintings than AI-generated paintings, *F*(1, 296) = 11.53, *p* = 0.001; and no difference was found for non-experts, *F*(1, 296) = 0.43, *p* = 0.515. Moreover, experts showed less liking toward AI-generated paintings than non-experts, *F*(1, 296) = 5.72, *p* = 0.017; and no difference was found for artist-made paintings, *F*(1, 296) = 0.04, *p* = 0.832.

**TABLE 2:** Mean values of liking ratings, purchase intention, and collection intention toward paintings.

Author × Art expertise			Painting style × Art expertise		
	Experts	Non-experts		Experts	Non-experts
**Liking rating**					
AI-generated	59.80 ± 2.21	67.36 ± 2.21	Western-style	67.12 ± 1.74	66.98 ± 1.66
Artist-made	70.54 ± 2.35	69.29 ± 2.15	Chinese-style	63.23 ± 1.81	69.66 ± 1.73
**Purchase intention**					
AI-generated	4.16 ± 0.14	4.46 ± 0.14	Western-style	4.58 ± 0.11	4.34 ± 0.10
Artist-made	4.75 ± 0.15	4.43 ± 0.13	Chinese-style	4.33 ± 0.11	4.56 ± 0.11
**Collection intention**					
AI-generated	4.29 ± 0.13	4.57 ± 0.13	Western-style	4.87 ± 0.11	4.50 ± 0.10
Artist-made	5.17 ± 0.14	4.68 ± 0.13	Chinese-style	4.59 ± 0.10	4.75 ± 0.10

The mean values and SDs in different conditions of the author and the art expertise interaction (left panel) and of the painting style and the art expertise interaction (right panel).

**FIGURE 3:**
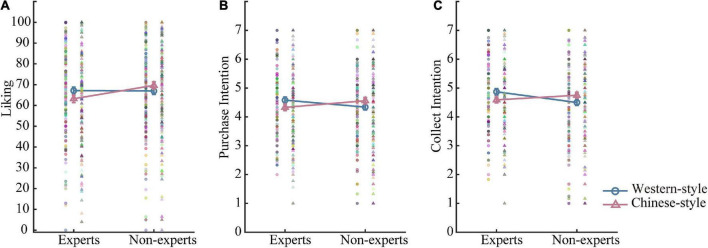
Painting style (Western style and Chinese style) by art expertise (experts and non-experts) interaction. Consistent results were found for liking rating **(A)**, purchase intention **(B)**, and collection intention **(C)**. Error bars stand for ± S.E.M.

The results of the painting style and the art expertise interaction are presented in Figure 3A, and mean values are listed in Table 2. Simple effect analysis showed that non-experts showed more liking toward Chinese-style paintings, *F*(1, 297) = 7.27, *p* = 0.007 than experts, but not for Western-style paintings, *F*(1, 297) = 0.01, *p* = 0.943. Experts showed more liking toward Western-style than Chinese-style paintings, *F*(1, 297) = 7.51, *p* = 0.007, and non-experts showed no preference in liking, *F*(1, 297) = 3.50, *p* = 0.062.

#### Purchase intention and collection intention of paintings

For purchase intention and collection intention, a 2 (art expertise: experts vs. non-experts) × 2 (author: AI vs. human artists) × 2 (style: Western vs. Chinese style) ANOVA indicated consistent results. The analysis revealed a significant main effect of the author effect [purchase intention: *F*(1, 295) = 3.95, *p* = 0.048, η*_*p*_*^2^ = 0.01; collection intention: *F*(1, 295) = 13.77, *p* < 0.001, η*_*p*_*^2^ = 0.05], a significant interaction effect of the author and the art expertise [purchase intention: *F*(1, 295) = 4.78, *p* = 0.029, η*_*p*_*^2^ = 0.02; collection intention: *F*(1, 295) = 8.38, *p* = 0.004, η*_*p*_*^2^ = 0.03], and a significant interaction effect of the style and the art expertise [purchase intention: *F*(1, 295) = 15.28, *p* < 0.001, η*_*p*_*^2^ = 0.05; collection intention: *F*(1, 295) = 21.71, *p* < 0.001, η*_*p*_*^2^ = 0.07]. Neither the main effect of the style, the main effect of the art expertise, nor the interaction effect of the style and the author, the interaction effect of the three factors were significant (*Fs* < 1.32, *ps* > 0.05).

For the interaction of the author and the art expertise, simple effect analysis on the purchase intention (Figure 2B) and collection intention (Figure 2C) revealed similar results. As expected, experts showed higher purchase and collection intentions toward artist-made paintings than AI-generated paintings (*Fs* > 8.36, *ps* < 0.005), and no difference was found for non-experts (*Fs* < 0.88, *ps* > 0.560). Besides, experts showed higher collection intention of artist-made paintings than non-experts, *F*(1, 296) = 5.36, *p* = 0.021; and no difference was found for AI-generated paintings, *F*(1, 296) = 2.12, *p* = 0.146. The purchase intention toward neither AI-generated paintings [*F*(1, 296) = 2.26, *p* = 0.134] nor artist-made paintings [*F*(1, 296) = 2.11, *p* = 0.148] was affected by art expertise.

For the interaction of the painting style and the art expertise, simple effect analysis on the purchase intention (Figure 3B) and collection intention (Figure 3C) also revealed similar results, except that experts were more willing to collect Western-style paintings than non-experts, *F*(1, 297) = 5.44, *p* = 0.020, but no difference in purchase intention. In addition, experts were more willing to buy and collect Western-style relative to Chinese-style paintings, *F*(1, 297) > 8.44, *p* < 0.004, while non-experts were more willing to buy and collect Chinese-style relative to Western-style paintings, *F*(1, 297) > 7.18, *p* < 0.008.

Collectively, art experts evaluated less favorably (less liking, lower purchase and collection intentions) AI-generated paintings relative to artist-made paintings, while non-experts showed no preference. Non-experts showed significantly higher purchase intention and collection intention toward Chinese-style paintings than Western-style paintings, but no difference in liking ratings, partially supporting H2b. Art experts evaluated AI-generated paintings lower than non-experts.

## Discussion

We investigated how AI art alters people’s liking, purchase intention, and collection intention toward Chinese-style and Western-style paintings, and whether art expertise plays a role. In study 1, several findings were revealed. One is that the main effect of the author under hypothesis H1a was not significant. Specifically, who made the art (AI vs. artists) would not influence evaluations, purchase intention, and collection intention toward paintings. Second, the main effect of painting style (ingroup preference) was revealed as hypothesized (H1b). Chinese participants favored Chinese-style paintings more than Western-style paintings. Third, although the interaction of the author and the style was not significant, we conducted *post-hoc* tests and found that participants preferred AI-generated Chinese-style paintings to AI-generated Western-style paintings in support of H1c. Study 2 further investigated the modulation effect of art expertise and found a significant main effect of the author, interaction of the author and the art expertise, and interaction of the style and the art expertise. In support of H2a, art experts evaluated less favorably (less liking, lower purchase and collection intentions) AI-generated paintings relative to artists-made paintings, while non-experts showed no preference. Non-experts showed significantly higher purchase intention and collection intention toward Chinese-style paintings than Western-style paintings, but no difference in liking ratings, partially supporting H2b. In support of H2c, art experts evaluated AI-generated paintings lower than non-experts. Overall, these findings partially supported our hypotheses.

We expected a bias against AI-generated paintings based on existing literature on the framing effect of labels or titles in empirical aesthetics (Kirk et al., 2009; Belke et al., 2010; Hawley-Dolan and Winner, 2011; Silveira et al., 2015; Mastandrea and Umiltà, 2016; Mastandrea and Crano, 2019). However, participants (non-experts) in study 1 showed no bias against AI-generated paintings. One explanation was that the label “AI-generated” might make observers feel novel (Israfilzade, 2020). Israfilzade (2020) found that abstract paintings were rated more novel and surprising when artificial intelligence accompanied the title, and no difference was found in terms of complexity, interestingness, and ambiguity arousal of the paintings. Moreover, participants in study 1 showed a preference for AI-generated Chinese-style to AI-generated Western-style paintings, in line with the uncertainty-identity hypothesis (Mastandrea et al., 2021). They might be uncertain about the AI-generated context and may resort to cultural identity as an art appreciation heuristic.

As expected, non-experts in this research (study 1 and study 2) showed a preference for Chinese-style relative to Western-style paintings, indicating the existence of ingroup bias in aesthetic evaluations (for a review, refer to Che et al., 2018). However, art experts in study 2 showed a preference for Western-style paintings. One explanation might be that people who are interested in art concur in their aesthetic judgments irrespective of their cultural backgrounds (Child, 1965; Iwao and Child, 1966; Iwao et al., 1969). This finding was consistent with results in Darda and Cross (2022a), which found that art experts tended to agree in their judgments and showed lower ingroup preference than non-experts.

Additionally, we expected that art expertise modulated the bias against AI-generated paintings. As expected, we found a bias among art experts but not non-experts, in line with Darda and Cross (2022b). However, this finding was inconsistent with Moffat and Kelly (2006) and Chamberlain et al. (2018), which indicated a bias against computer-generated artworks by both experts and non-experts. One explanation for this discrepancy might be the stimuli adopted. We used artist-made paintings and labeled them as made by AI or artists. Chamberlain et al. (2018) selected paintings from computer art databases and matched them with man-made counterparts. The paintings used in our studies were of high artistic value, meanwhile avoiding being too well-known to be recognized by participants. Therefore, it is important to note that these findings should only be interpreted to the current image set and should not be broadened to the overall comparison of AI-generated and artist-made paintings.

### Implications and limitations

As stated in Leder et al. (2012), “Art is a unique feature of human experience. It involves the complex interplay among stimuli, persons, and contexts.” This may explain why the aesthetic appreciation of experts and non-experts differs to a great extent, and why the author of artworks matters to experts. The findings in this study offer support for the bias against AI-generated paintings and the modulation effect of art expertise, contributing to the framing effect and ingroup bias research in empirical aesthetics. In terms of applications, our findings also suggest that AI-related personnel, such as designers of websites and apps taking AI art as a focus, should consider how to decrease potential users’ bias against AI-generated paintings as well as enrich painting styles to meet individuals’ tastes and preferences. Increasing anthropomorphism of the “AI” system might be useful. Previous evidence suggested that viewing the creation of artwork by a robot increased aesthetic appreciation for it (Chamberlain et al., 2018). It is worth noting that perceptions of AI anthropomorphicity can be manipulated by changing the language used to talk about AI—as a tool vs. agent (Epstein et al., 2020). AI-enhanced, rather than AI-generated, has been used in the research report, and it is essential to emphasize that AI/machine was dedicated to helping unlock human creative potential (Ploin et al., 2022).

Several limitations in this research should be addressed in future studies. First, the sample we recruited may have restricted the generalization of findings in the current studies. For ease of sampling, we collected data mainly from students and teachers in design and art education in China. Famous artists and a larger size of sample would be more appropriate. In addition, we only recruited Chinese participants for this research. It is preferable to recruit participants from both China and Western culture in future studies. Second, some relevant characteristics were not collected prior to the studies, such as the participants’ level of familiarity with Western-style and Chinese-style paintings, making it difficult to perform assessments of the specific effects of familiarity with paintings. The inclusion of characteristics such as this would add value to analyses in future studies. Third, although we conducted a pilot to make sure these paintings would not be recognized (author and name of the painting), especially by our sample population, several teachers reported that they might see the painting before even though they could not recall its name. Asking participants whether they recognized any of the paintings at the end of the study would be a better way to exclude the confounding effect.

## Data availability statement

The data generated during and/or analyzed during the current study are available from the corresponding authors on reasonable request.

## Ethics statement

Ethical review and approval were not required for the study on human participants in accordance with the local legislation and institutional requirements. Participants provided online informed consent before their enrollment in the study.

## Author contributions

LG: conceptualization, methodology, formal analysis, writing (original draft), and visualization. YL: conceptualization and writing (revision). Both authors contributed to the article and approved the submitted version.

## Footnotes

1
https://www.wjx.cn/
